# Supplementary material for: Prognostic and clinicopathological role of RACK1 for cancer patients: a systematic review and meta-analysis
Source: PeerJ. 2023 Aug 14;11:e15873. doi: 10.7717/peerj.15873 (PMC10434108; doi:10.7717/peerj.15873)
Supplement: Table S2 [file peerj-11-15873-s002.docx]

**Table S2: Detailed search strategy for each database.**

| Databases | Steps | Strategies |
| --- | --- | --- |
| PubMed | #1 | ((((Receptor of Activated C Kinase 1[All Fields]) OR (RACK1[All Fields])) OR (RACK1 Protein[All Fields])) OR (GNB2L1[All Fields])) |
|  | #2 | (((((((((Neoplasms[MeSH Terms]) OR (Tumor[All Fields])) OR (Neoplasm[All Fields])) OR (Tumors[All Fields])) OR (Neoplasia[All Fields])) OR (Neoplasias[All Fields])) OR (Cancer[All Fields])) OR (Cancers[All Fields])) OR (Malignant Neoplasm[All Fields])) |
|  | #3 | #1 AND 2# |
| Embase | #1 | 'neoplasm'/exp OR neoplasms OR tumor OR neoplasm OR tumors OR neoplasia OR neoplasias OR cancer OR cancers |
|  | #2 | 'receptor of activated c kinase 1'/exp OR 'receptor of activated c kinase 1' OR 'rack1 protein' OR gnb2l1 OR rack1 |
|  | #3 | #1 AND #2 |
| Web of Science | #1 | TS=(neoplasms OR tumor OR neoplasm OR tumors OR neoplasia OR neoplasias OR cancer OR cancers OR "Malignant Neoplasm" ) |
|  | #2 | TS=("receptor of activated c kinase 1" OR "rack1 protein" OR "gnb2l1" OR "rack1" ) |
|  | #3 | #1 AND #2 |
| Scopus | #1 | TITLE-ABS-KEY ( "receptor of activated c kinase 1" OR "rack1 protein" OR "gnb2l1" OR "rack1") |
|  | #2 | TITLE-ABS-KEY ( neoplasms OR tumor OR neoplasm OR tumors OR neoplasia OR neoplasias OR cancer OR cancers OR "Malignant Neoplasm" ) |
|  | #3 | LIMIT-TO ( DOCTYPE , "ar" ) |
|  | #4 | #1 AND #2 AND #3 |
| Cochrane Library | #1 | MeSH descriptor: [Neoplasms] explode all trees |
|  | #2 | neoplasms OR tumor OR neoplasm OR tumors OR neoplasia OR neoplasias OR cancer OR cancers OR "Malignant Neoplasm" |
|  | #3 | MeSH descriptor: [Receptors for Activated C Kinase] explode all trees |
|  | #4 | "receptor of activated c kinase 1" OR "rack1 protein" OR "gnb2l1" OR "rack1" |
|  | #5 | #1 OR #2 |
|  | #6 | #3 OR #4 |
|  | #7 | #5 AND #6 |
