# Supplementary material for: Prognostic and clinicopathological role of RACK1 for cancer patients: a systematic review and meta-analysis
Source: PeerJ. 2023 Aug 14;11:e15873. doi: 10.7717/peerj.15873 (PMC10434108; doi:10.7717/peerj.15873)
Supplement: Table S3 [file peerj-11-15873-s003.docx]

**Table S3. Critical appraisal of included studies in this** **systematic review and meta-analysis.**

| Studies | S1 | S2 | S3 | S4 | C1 | O1 | O2 | O3 | Total scores |
| --- | --- | --- | --- | --- | --- | --- | --- | --- | --- |
| Cao, X. X. 2009 | ★ | ★ | ★ | ★ | ★ | ★ | ★ | ★ | 8 |
| Nagashio, R. 2010 | ★ | ★ | ★ | ★ | ★ | ★ |  | ★ | 7 |
| Shi, S. 2012 | ★ | ★ | ★ | ★ | ★ | ★ |  | ★ | 7 |
| Zhong, X. 2013 | ★ | ★ | ★ | ★ | ★ | ★ | ★ | ★ | 8 |
| Jin, S. 2014 | ★ | ★ | ★ | ★ | ★ | ★ |  | ★ | 7 |
| Lin, Y. 2014 | ★ | ★ | ★ | ★ | ★ | ★ |  | ★ | 7 |
| Chen, L. 2015 | ★ | ★ | ★ | ★ | ★ | ★ | ★ | ★ | 8 |
| Wang, N. 2015 | ★ | ★ | ★ | ★ | ★ | ★ | ★ | ★ | 8 |
| Li, X. 2016 | ★ | ★ | ★ | ★ | ★ | ★ | ★ | ★ | 8 |
| Lv, Q. L. 2016 | ★ | ★ | ★ | ★ | ★ | ★ |  | ★ | 7 |
| Peng, H. 2016 | ★ | ★ | ★ | ★ | ★ | ★ |  | ★ | 7 |
| Liu, C. 2017 | ★ | ★ | ★ | ★ | ★ | ★ |  | ★ | 7 |
| Qu, G. 2017 | ★ | ★ | ★ | ★ | ★ | ★ | ★ | ★ | 8 |
| Han, H. 2018 | ★ | ★ | ★ | ★ | ★ | ★ | ★ | ★ | 8 |
| Liu, S. 2018 | ★ | ★ | ★ | ★ |  | ★ | ★ | ★ | 7 |
| Xiao, T. 2018 | ★ | ★ | ★ | ★ | ★ | ★ | ★ | ★ | 8 |
| Li, X. Y. 2019 | ★ | ★ | ★ | ★ | ★ | ★ |  | ★ | 7 |
| Zhang L 2019 | ★ | ★ | ★ | ★ | ★ | ★ | ★ | ★ | 8 |
| Shen, C. 2020 | ★ | ★ |  | ★ | ★ | ★ |  | ★ | 6 |
| Wu, H. 2020 | ★ | ★ | ★ | ★ | ★ | ★ |  | ★ | 7 |
| Yu, Z. 2021 | ★ | ★ | ★ | ★ | ★ | ★ | ★ | ★ | 8 |
| Xu, L. 2022 | ★ | ★ | ★ | ★ | ★ | ★ | ★ | ★ | 8 |

S1: Representativeness of the exposed cohort

S2: Selection of the non-exposed Cohort

S3: Ascertainment of exposure

S4: Demonstration that outcome of interest was not present at start of study

C1: Comparability of cohorts on the basis of the design or analysis

O1: Assessment of outcome

O2: Was follow-up long enough for outcomes to occur

O3: Adequacy of follow up of cohorts
