# Supplementary figures and images for: Prognostic and clinicopathological role of RACK1 for cancer patients: a systematic review and meta-analysis
Source: PeerJ. 2023 Aug 14;11:e15873. doi: 10.7717/peerj.15873 (PMC10434108; doi:10.7717/peerj.15873)

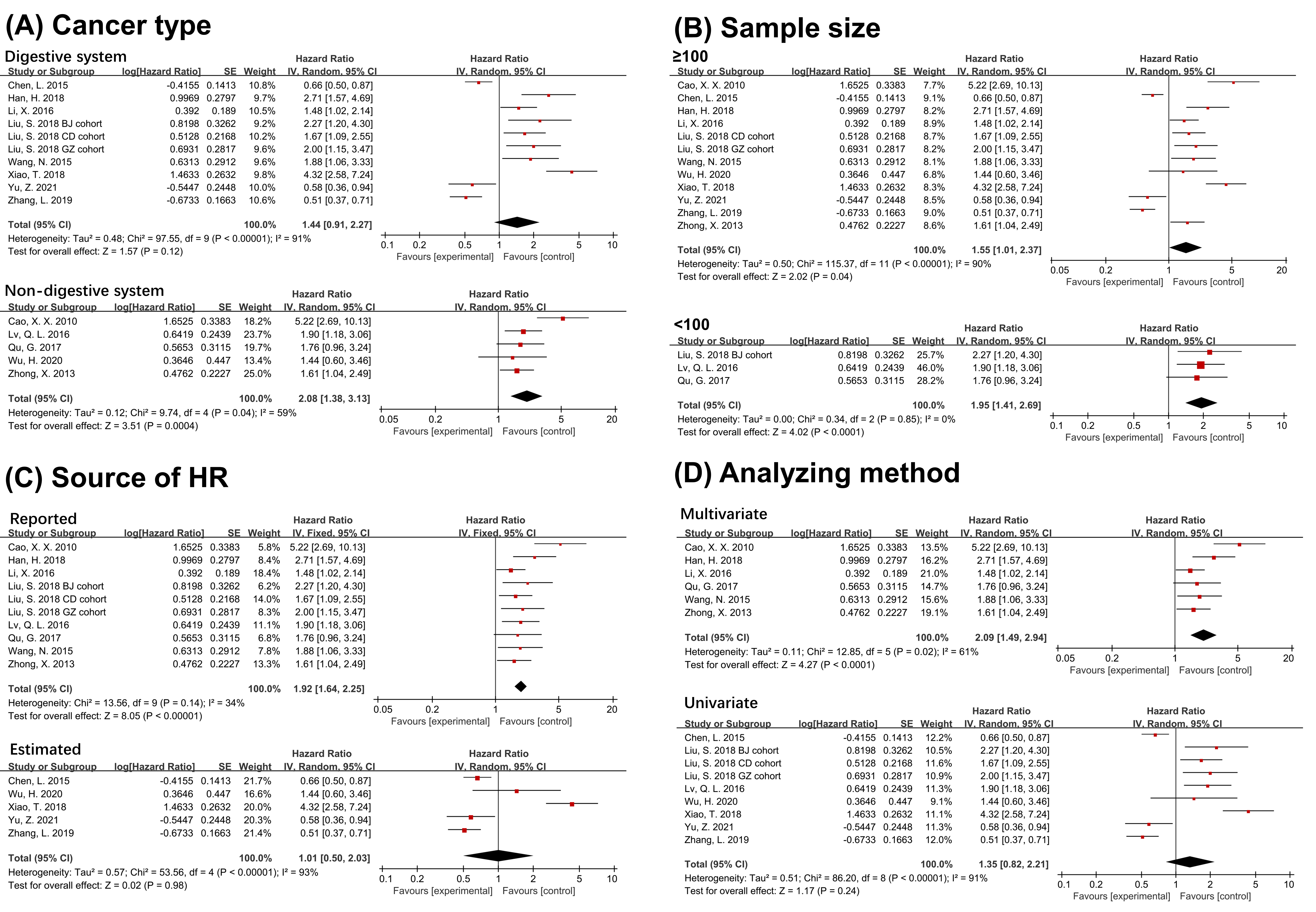

Supplement: Figure S2 [file peerj-11-15873-s005.png]
